# Endovascular Treatment of Intracranial Atherosclerotic Stenosis: Current Debates and Future Prospects

**DOI:** 10.3389/fneur.2018.00666

**Published:** 2018-08-21

**Authors:** Jichang Luo, Tao Wang, Peng Gao, Timo Krings, Liqun Jiao

**Affiliations:** ^1^Department of Neurosurgery, Xuanwu Hospital, Capital Medical University, Beijing, China; ^2^UHN Joint Department of Medical Imaging Division of Neuroradiology, Toronto Western Hospital, University of Toronto, Toronto, ON, Canada

**Keywords:** intracranial atherosclerotic stenosis, endovascular treatment, patient selection, angioplasty, stent, operator experience

## Abstract

Intracranial atherosclerotic stenosis (ICAS) is a common cause of transient ischemic attack (TIA) and ischemic stroke. Endovascular treatment, including balloon angioplasty alone, balloon-mounted stents, and self-expandable stent placement with or without prior angioplasty, is an alternative to medical treatment for the prevention of recurrent TIA or ischemic stroke in patients with ICAS. Although the SAMMPRIS and VISSIT trials supported medical management alone against endovascular treatments, both randomized controlled trials (RCT) were criticized due to flaws relating to patient-, intervention-, and operator-related factors. In this review, we discuss the current debate regarding these aspects and suggest approaches to solve current controversies in the future. In our opinion, endovascular treatment in carefully selected patients, individualized choice of endovascular treatment subtypes, and an experienced multidisciplinary team managing the patient in the pre-, peri- and post-procedural period have the potential to provide safe and efficious treatment of patients with symptomatic ICAS.

## Introduction

Stroke is the second leading cause of death worldwide, after ischemic heart disease (1, 2), and 87% of cases are ischemic stroke (3). Intracranial atherosclerotic stenosis (ICAS) is one of the most common causes of ischemic stroke, accounting for up to 30 to 50% of ischemic stroke in Asia (4). According to the report titled “Global Burden of Stroke,” the incidence and prevalence of stroke has increased gradually in developing countries, which bear most of the burden caused by stroke across the world (5).

To date, medical management, including antiplatelet therapy, intensive cardiovascular risk factor control, as well as lifestyle management, is still recommended as the first-line therapy for ICAS to prevent recurrent transient ischemic attack (TIA) and ischemic stroke. However, despite intensive medical management, a high risk of recurrent TIA and stroke was still observed in patients with high-grade (70–99%) symptomatic ICAS. This group of patients was considered to be refractory to aggressive medical therapy. Data from the SAMMPRIS trial (Stenting and Aggressive Medical Management for Preventing Recurrent Stroke in Intracranial Stenosis trial) showed that the rate of 1 year stroke or death in symptomatic ICAS patients with more than a 70% degree of stenosis was as high as 12.6% in the medical arm (6). In addition, a lifestyle coach was assigned to every patient in the medical arm, which is unlikely to be available within general healthcare systems, especially in low- or middle-income countries (7). Therefore, endovascular treatment, including balloon angioplasty alone, balloon-mounted stent placement, or self-expandable stent placement, was considered as an alternative option for the prevention of recurrent TIA or ischemic stroke in patients with a high degree of ICAS.

Although the results of both the SAMMPRIS and VISSIT (the Vitesse Intracranial Stent Study for Ischemic Stroke Therapy) trials supported the use of aggressive medical management as being superior to stent therapy (6, 8), some prospective and retrospective studies from both Europe and Asia reported encouraging results for endovascular treatment (9–14). In this article, we will review the current literature related to angioplasty or stent placement for ICAS and discuss the current debate regarding three aspects: patient-, intervention-, and operator-related factors. We will also discuss future research directions for dealing with current controversies and how to solve them.

## Three aspects of factors affect outcomes of endovascular treatment

Endovascular treatment of symptomatic ICAS has been facing controversy since the publication of the SAMMPRIS trial, which was the only multicenter, prospective, randomized controlled trial (RCT) of intracranial stenting for ICAS in America, enrolling symptomatic patients with recent (i.e., within 30 days) TIA or non-disabling stroke and who were identified as having 70–99% stenosis of a major intracranial artery. The aim of the trial was to compare the efficiency of recurrent stroke prevention between percutaneous transluminal angioplasty and stenting (PTAS) with aggressive medical management vs. aggressive medical management alone. The initial design was to recruit 764 patients randomly divided into the PTAS group or medical management group. However, the trial was halted early because of the unexpected result of a 30 days death or stroke rate of 14.7% (10.2% ischemic and 4.5% hemorrhagic) in the PTAS group, compared with 5.8% in the medical management group. This result indicated that the short-term safety of medical management was superior to PTAS in the patients treated in this trial. Moreover, the long-term efficacy of medical management in the SAMMPRIS trial was also superior to PTAS, with 1, 2, and 3 years rates of mortality or stroke of 12.6, 14.1, and 14.9% in the medical management group compared to 19.7, 20.6, and 23.9% in the PTAS group, respectively (7). Similar to the SAMMPRIS trial, the results of the VISSIT trial, published in 2015, which was the first randomized clinical trial comparing balloon-mounted stent treatment with medical therapy in patients with severe stenosis (70–99%) of symptomatic ICAS, also indicated that aggressive medical management was superior to angioplasty or stenting. The 30 days and 1 year TIA or stroke rates were 24.1 and 36.2% in the stent group vs. 9.4 and 15.1% in medical group, respectively. Because of the negative results of stenting from both RCTs, the attitude toward angioplasty or stenting to prevent recurrent TIA or stroke caused by intracranial atherosclerosis has diminished the enthusiasm for angioplasty or stenting for treatment of intracranial atherosclerosis in most centers (15, 16). However, controversies have been raised for both trials including patient-, intervention-, and operator-related factors. These may influence the outcomes of PTAS for the treatment of ICAS. Therefore, there are still ongoing debates focusing on the best treatment of ICAS.

### Patient selection

The method of patient selection is one of the major criticisms of the SAMMPRIS trial. There are three primary points to follow when enrolling patients in a trial, which were ignored in SAMMPRIS.

First, more than one-third (35.3%) of the patients in the PTAS group were not refractory to medical therapy when qualifying events were evaluated for enrollment in SAMMPRIS. However, Wingspan stent, a self-expanding nitinol intracranial stent and the only type of stent used in SAMMPRIS, was approved for Humanitarian Device Exemption in 2005 and recommended for use by the Food and Drug Administration (FDA) only in symptomatic patients with more than 50% intracranial stenosis after failure of antithrombotic therapy (17). Patients who failed antithrombotic therapy may benefit more from endovascular treatment than those who did not. A study that included symptomatic ICAS patients, with 95.5% (43/45) failing at least one kind of antithrombotic therapy, showed a 30 days stroke or vascular death rate of 6.6% after endovascular treatment, which was significantly better than SAMMPRIS (18).

Second, the median time from the qualifying event to randomization in SAMMPRIS was 7 days (interquartile range: 4–16 days) in the PTAS group, which indicated that most patients were treated in the acute or subacute stage. The detailed analysis of SAMMPRIS results demonstrated no relationship between the time from the qualifying event to PTAS and the risk of ischemic events (19). Early recanalization of intracranial stenosis with PTAS may rescue the ischemic penumbra by increasing the downstream flow of the territory at the stenosis artery, which may improve symptoms of neurologic deficits (20). However, the problems of stability of plaque and reperfusion hemorrhage in the acute or subacute stage must be considered. A high risk of recurrent TIA or stroke due to the “snow-plowing” of unstable plaque was regarded as the major cause of perforator infarction in patients treated with endovascular therapy (21). A *post-hoc* analysis of periprocedural strokes in patients who underwent angioplasty or stent placement in the SAMMPRIS trial found that perforator occlusion was the most common cause of periprocedural stroke (19). Another risk of emergency endovascular treatment is reperfusion hemorrhage. The intracranial microcirculation in the territory of acute cerebral infarction is considered to be unstable, which may result in a higher risk of reperfusion hemorrhage after the procedure for symptomatic ICAS patients treated in the acute or subacute stage. In a surveillance study, the Japanese Registry of Neuroendovascular Therapy, 1,133 ICAS patients underwent intracranial percutaneous transluminal angioplasty or stenting. The results showed that the number of hemorrhagic complications was significantly greater in patients who received endovascular treatment between 24 h and 14 days after the onset of symptoms as compared to those who received treatment later (22). Hence, PTSA implemented at the proper time may decrease the risk of perioperative complications.

**Figure 1:**
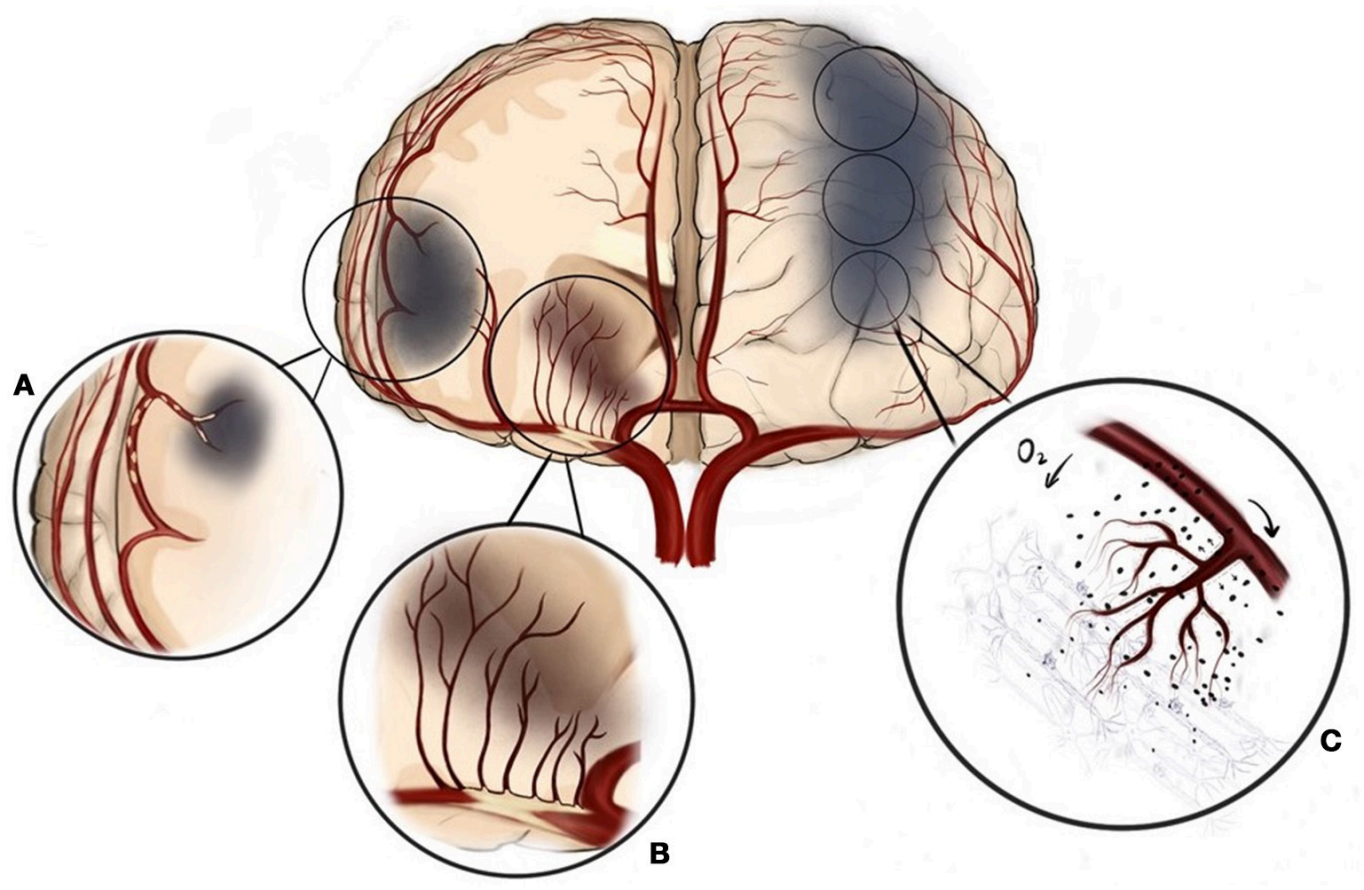
Three different mechanisms of ischemic stroke can be present in intracranial atherosclerosis. **(A)** Artery to artery embolism, **(B)** Local perforator ischemia, **(C)** Hemodynamic hypoperfusion.

Third, the mechanism of ischemic stroke was not reported upon and may dramatically effect upon the complication and efficacy rate of intracranial stenosis. Intracranial atherosclerotic disease may become symptomatic due to (a) local perforator ischemia, (b) artery to artery embolism, (c) hemodynamic hypoperfusion, or, (d) a combination of the aforementioned mechanisms (23–25) (see Figure 1). Efficacy and risks of treatment will naturally differ between each different pathological mechanism. Studies found that symptomatic ICAS patients with hypoperfusion or poor collateral circulation in the downstream area of stenotic arteries could benefit more from endovascular treatment than those with other mechanisms of ischemic stroke (26). A study found that a combination of dual antithrombotic medicine, high-dose statins, and rigorous lifestyle management may be effective for lowering the risk of artery-to-artery embolism in patients with ICAS (25). Moreover, perforator ischemia possibly causes an excessive risk of periprocedural stroke due to occlusion of (additional) perforators through “snow-plowing” plaque toward their origins (27). Abou-Chebl et al. found that the exclusion of symptomatic patients with perforator infarction before PTAS in SAMMPRIS could decrease the rate of 30 days ischemic stroke from 14.7 to 9.4% (28). Therefore, identification of the mechanism underlying the recent ischemic stroke event may be a way to reduce the risk of perioperative complications of PTAS treatment. However, no further classification based on the specific mechanisms of stroke was made at the enrolment of the SAMMPRIS trial. Patients were simply grouped as TIA or stroke, which could not differentiate patients with hypoperfusion or poor collateral circulation downstream of the stenotic arteries.

The VISSIT trial had similar flaws. It was impossible to tell whether the participants failed antithrombotic therapy or not. The median time from qualifying events to stenting was 12.3 days, also within the acute or subacute stage. Hemodynamic symptomatology was not used to select participants (8).

In summary, the aforementioned flaws of study design in both the SAMMPRIS and VISSIT trials have a non-negligible impact on the credibility of previous studies to deny the potential positive effect in carefully selected patients with symptomatic ICAS for PTAS.

Contrary to SAMMPRIS and VISSIT, results from several prospective trials in Asia demonstrated promising outcomes concerning endovascular treatment for ICAS: A multicenter prospective study in China included 354 symptomatic high-grade ICAS patients with hypoperfusion symptoms and poor collaterals. These patients received balloon-mounted stent, self-expandable stent placement, or balloon angioplasty alone based on technical considerations regarding access and lesion morphology in the subacute phase after the onset of symptoms; patients with embolic thrombosis, lacunar infarcts, severe vascular tortuosity, non-atherosclerotic lesions, or a baseline modified Rankin Scale (mRS) score of >3 were excluded. The 30 days stroke, TIA, or death rate was 4.3%, which was significantly lower than that in the SAMMPRIS and VISSIT trials (12). Prior to this study, a single-center prospective study with 158 symptomatic ICAS patients and the same inclusion criteria and endovascular treatment demonstrated a 30 days composite stroke, myocardial infarction, or death rate of 4.4% (11). Similarly, another investigator-initiated, government-funded, prospective, multicenter registration trial with 100 symptomatic ICAS patients 3 weeks after the index ischemic event and without perforator stroke and/or disabling stroke (mRS >3) who were treated with angioplasty and self-expandable stent demonstrated an overall 30 days stroke and/or death rate of 2% (95% confidence interval, 0.2–7.0%) (13). Hence, the selection of patients and the timing of treatment appears to be of importance for ICAS.

### The type of treatment

Treatment type may also affect outcomes. In SAMMPRIS, there were 224 lesions of ICAS, among which 61.2% were in the anterior circulation and the remaining were in the posterior circulation. The sole endovascular stent was the Wingspan stent. However, data showed that complication rates were different between the anterior and posterior circulation. Data from a systematic review including 31 studies and 1,177 symptomatic, high-grade ICAS patients receiving stent treatment demonstrated a significantly lower rate of periprocedural complications in the anterior circulation than that in the posterior circulation (6.6 vs. 12.1%, *P* < 0.01) (29). There are many types of endovascular techniques available for ICAS treatment, including balloon angioplasty alone, balloon-mounted stent (Pharos Vitesse), and self-expandable stents (Wingspan), each of which has its own features and specific advantages relating to different intracranial artery lesions. The characteristics and location of these lesions can be used to choose the type of angioplasty or stenting to treat symptomatic ICAS patients:

### Balloon angioplasty alone

Balloon angioplasty alone is the first and simplest endovascular therapy used for the treatment of intracranial stenosis, which increases perfusion of the downstream territory of the stenotic artery by dilating the caliber of the stenotic segment, decreasing or eliminating ongoing or recurrent neurologic symptoms, and potentially delaying or preventing secondary occlusion and stroke. The enthusiasm of balloon angioplasty alone for intracranial stenosis can be traced to the 1980s, when the prognosis of high-grade intracranial stenosis was poor with medical treatment (30, 31). Sundt et al. reported the first successful cases of transluminal balloon angioplasty for patient with high-grade, atherosclerotic, stenotic basilar artery who were refractory to anticoagulant therapy, of which the angiographic and short-term clinical results were excellent (32). Unfortunately, with more cases of intracranial stenosis with balloon angioplasty reported, more periprocedural complications were also reported, such as arterial dissection with consecutive thrombosis or rupture, residual stenosis due to sequestration or vessel recoiling, and acute or subacute vascular occlusion due to the formation of a wall hematoma (33). The high risk of complications induced the development of new technologies of balloon angioplasty. Submaximal balloon angioplasty with slow inflation was developed and recommended as a proper option for intracranial stenosis (33). In a study of 41 consecutive, symptomatic, high-grade (≥70%) ICAS patients, treatment was submaximal balloon angioplasty alone. The 30 days event rate and 1 year perioperative and ischemic event-free survival rate were 4.9 and 91%, respectively, both of which were better than those of the medical and PTAS group in the SAMMPRIS trial (34). Recently, a prospective phase I trial of 24 patients with significant intracranial stenosis treated with submaximal balloon angioplasty alone also reported better safety outcomes, with no 30 days ischemic stroke in the territory of the treated stenotic vessel and good efficacy outcomes, including a 1 year recurrent stroke rate of 5.55% and no mortality or hemorrhage event (35). Therefore, using current techniques and equipments, intracranial balloon angioplasty alone can be performed safely and efficiently in patients with symptomatic ICAS.

### Balloon-mounted stent

In the early stages of intracranial stent deployment, most balloon-mounted stents used to treat symptomatic ICAS patients were coronary stents, which were not designed for intracranial vasculature and thus were difficult to deliver through the tortuous cervical and intracranial vasculature (36). Therefore, the deployment of a balloon-mounted stent often resulted in distortion of the regional anatomy and sometimes led to traumatic injury to the tortuous vascular segments because of the stiffness and the lack of conformability of the high-pressure double-lumen balloon catheter. Meanwhile, a balloon-mounted stent demands a greater expansion to inflate the lumen but lacks intrinsic expansion forces, which may increase the risk of perforator damage due to plaque shifting when the lesion is near or in the location of perforating arteries (37). With the advancement of intracranial stents, various types of intracranial balloon-mounted stents have been developed for the treatment of symptomatic ICAS (38). Although some inherited flaws of intracranial balloon-mounted stents still exist, they have advantages in some aspects. First, the stenosis can be inflated in a single step by a single operator. Second, the radial force of current used is strong enough to withhold the recoil phenomenon generated by the plaque or the vessel wall. Third, the likelihood of exact stent placement is improved, which keeps the stent length short and avoids covering the normal vessel segment (39). The INTRASTENT registry study comparing balloon-mounted stents to self-expandable stents in 409 symptomatic ICAS patients reported no statistically significant difference in complication rates, but the balloon-mounted stent was prone to a higher risk of perforator strokes, while the self-expandable stent tended to result in more thromboembolic events (40). A recent study comparing the short-term outcomes of stenting in 97 patients with symptomatic intracranial vertebrobasilar artery stenosis showed that balloon-mounted stents have a lower rate of residual stenosis and are more suitable for the patient with smooth arterial access and a short and concentric stenosis (Mori A lesion) than the self-expandable stent (41). Given that the residual stenosis rate was one of the major factors affecting restenosis, less residual stenosis is thus more beneficial for the prevention of restenosis (42). In summary, these advantages of the balloon-mounted stent show its potential for the treatment of symptomatic ICAS.

### Self-expandable stent

The only currently available self-expandable stent (Wingspan) has been the most widely used intracranial stent for the treatment of ICAS ever since its FDA's approval. In clinical practice, submaximal balloon angioplasty is performed prior to deployment of the stent (38). Because the Wingspan stent system is more flexible and passes the tortuous intracranial vasculature more easily than balloon-mounted systems, it has a higher technical success rate (39). Moreover, the Wingspan stent system has a lower risk of perforator infarctions (40), because the angioplasty can be undersized thus, minimizing the risk to the adjacent normal parent vessel. In addition, the small outward radial force of the self-expandable stent decreases the compression force delivered to the plaque near the perforating arteries (17). These advantages of the self-expandable stent have made its use more prevalent in the treatment of ICAS. However, the design of the self-expandable stent still has considerable flaws. On one hand, its two-step maneuver may lengthen procedure duration, which potentially increases the risk of embolic stroke. On the other hand, the exchange wire maneuver may increase the risk of subarachnoid hemorrhage due to inadvertent and uncontrolled movement of guide-wire tip (36). Regarding the restenosis rate, the self-expandable stent is inferior to the balloon-mounted stent. A study of 46 lesions in 45 symptomatic ICAS patients who had failed antithrombotic therapy were treated with the Wingspan stent. Results showed that the restenosis rate (defined as more than 50% of the initial lumen) was 42.8, 9.5% of which was symptomatic (18).

To our best knowledge, contemporary angioplasty and stenting used to treat symptomatic ICAS patients has some specific advantages for different lesions of ICAS. The individualized selection of different subtypes of PTAS for patients may influence the outcome of the treatment.

### Operator experience

Operator experience was shown to be a factor related to the outcome of endovascular therapy for ICAS: greater operator experience was associated with a lower rate of perioperative complications (43, 44). However, a *post-hoc* analysis of the SAMMPRIS trial showed that operators with more credentialing case numbers (>10) were associated with higher 30 days complication rates compared to those with fewer cases (19.0 vs. 9.9%, *P* = 0.11), whereas high-volume centers with enrollment ≥12 had lower rates of hemorrhagic stroke compared with low-volume centers (2.7 vs. 9.8%, *P* = 0.043) (45). This controversial result showed no correlation between operator experience and the volume of centers, and more experienced operators were prone to having more periprocedural complications. Data from the National Institutes of Health Multicenter Wingspan Registry showed that operators in high-volume centers were more proficient and had lower complication rates than those in low-volume centers (46). Hence, the credibility of using 10 cases (as used in SAMMPRIS) to assess the experience of operators is questionable. Ten cases could have been underestimated for assessing the adequacy of experience with the Wingspan procedure (47). In addition, operators enrolled in SAMMPRIS were required to submit 20 cases of intracranial angioplasty, but only three cases had to be with the Wingspan system (48). Therefore, different designs of the Wingspan stent and other types of stents, as well as the tortuous vasculature in atherosclerotic lesions, may account for the insufficient credentialing criteria of three cases of Wingspan stent system (28).

## Prospect in future

Endovascular therapy with careful selection of patients, proper types of PTAS, and experienced operators may reduce the risks of perioperative complications and provide greater benefit for symptomatic ICAS patients. Therefore, we should pay more attention to these aspects in the future.

### Careful selection of patient

It is important to carefully select applicable patients with symptomatic intracranial stenosis, which is the first step in reducing the rate of perioperative complications.

ICAS patients who fail under medical management may require endovascular treatment. In 2012, the FDA modified the indication of the Wingspan stent, highlighting that patients identified as refractory to medical management must meet the criteria of having at least two strokes while receiving aggressive medical management (49). Patients who are refractory to medical management, defined as a recurrent ischemic event despite the combination of maximal-dose dual antiplatelet therapy, intensive cardiovascular risk factor control, and rigorous lifestyle management, may benefit more from endovascular treatment.

**Figure 2:**
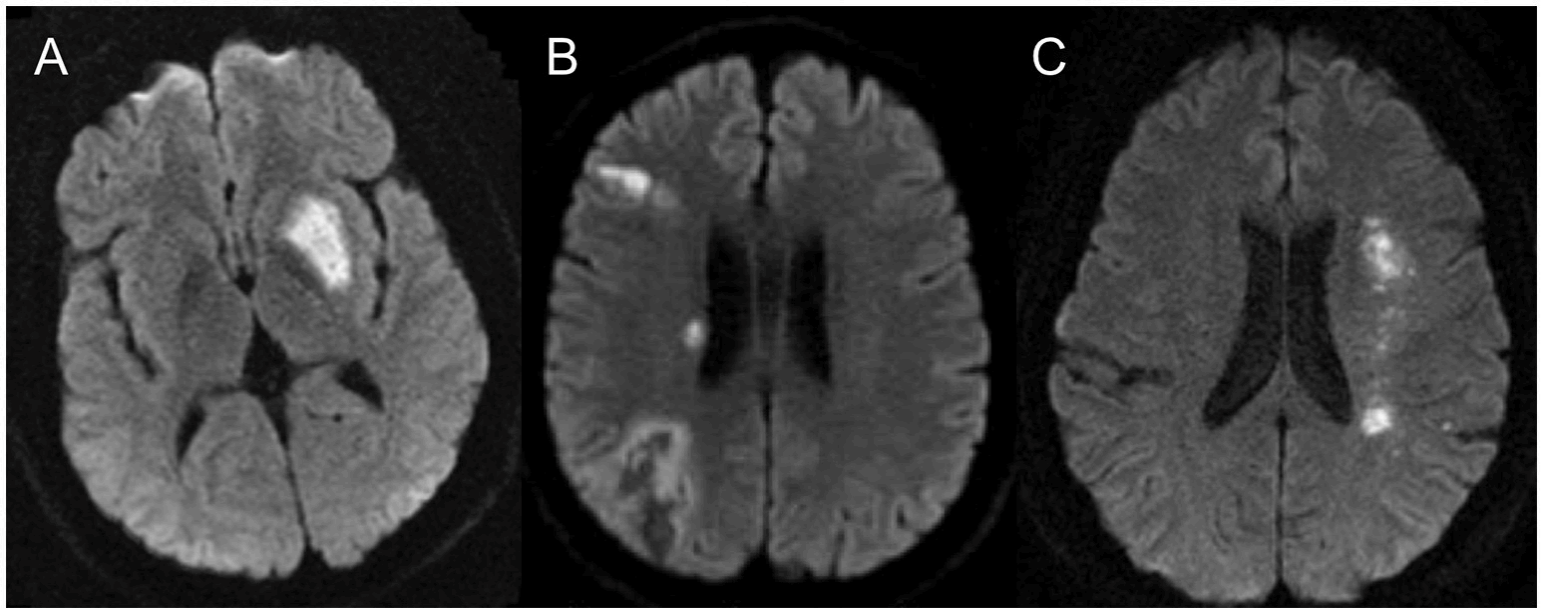
Ischemia pattern for the different pathological mechanisms seen in intracranial atherosclerosis: **(A)** demonstrates the typical perforator type infarct, **(B)** demonstrates the artery to artery embolic infarct and **(C)** demonstrates the classical deep watershed zone for the hemodynamic infarcts.

**Figure 3:**
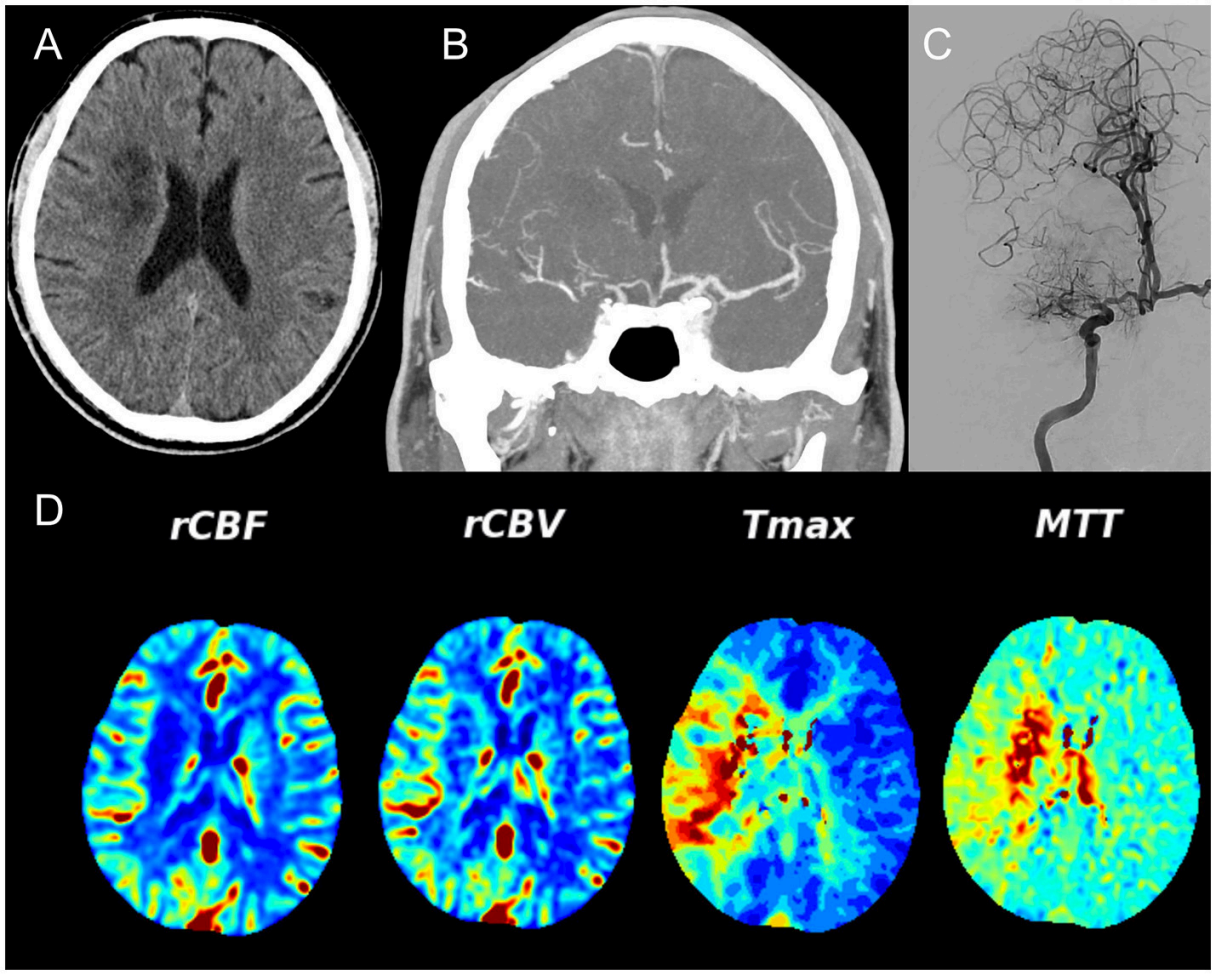
Unenhanced CT **(A)** demonstrates recent ischemia in a deep watershed pattern, CTA **(B)** in a coronal view shows a high degree MCA stenosis that is confirmed on conventional angiography **(C)**. Note the excellent collateral network from the leptomeningeal collaterals from the ACA territory toward the MCA territory. **(D)** Demonstrates the CT Perfusion parameters – relative cerebral blood flow (rCBF) and — volume (rCBV) that are both still normal whereas the Time to maximum contrast (Tmax) and the mean transit time (MTT) are significantly delayed over the right hemisphere indicating hypoperfusion.

In addition, identification of the mechanism for the recent stroke is also important. Patients with hypoperfusion or poor collateral circulation of the downstream territory at the stenotic arteries may benefit more from endovascular treatment, while patients with perforator occlusion will have no benefit (or may even be harmed) from angioplasty. Patients with artery-artery embolism may benefit from aggressive medical management. In clinical practice, advanced neuroimaging technologies could be used to identify stroke mechanisms. For instance, brain imaging of infarct patterns on diffusion-weighted imaging could infer the underlying stroke mechanisms (50). The perforator pattern is characterized by infarct lesions in the subcortical or perforator territory, i.e., in the territory perfused by perforating vessels that originate at the site of stenosis. An arterial embolic pattern is characterized by infarct lesions located in the downstream territory of the stenotic vessel (cortical, subcortical, or both) and is limited to the territory supplied by a single intracranial culprit artery. The border-zone or hemodynamic pattern is characterized by one or more infarct lesions located in the internal border-zone region in the corona radiata or centrum semiovale and/or in the cortical border-zone region, between the middle cerebral artery and anterior cerebral artery or between the middle cerebral artery and posterior cerebral artery (see Figure 2 for examples). The mixed pattern presents a combination of any of the previous infarct patterns described above (51–53). Hypoperfusion may also be estimated by reduced blood flow on computed tomography perfusion, perfusion-weighted imaging on magnetic resonance or single-photon emission computed tomography (see Figure 3) (54). Collaterals may be assessed on digital subtraction angiography with the American Society of Interventional and Therapeutic Neuroradiology/Society of Interventional Radiology Collateral Flow Grading System, which could be categorized as none (grade 0), poor (grades 1 or 2), or good (grades 3 or 4) (55).

Moreover, optimizing the time of endovascular treatment from qualifying events is also important to reduce procedural-related complications, including plaque detachment and reperfusion hemorrhage. Several studies demonstrated that the early time period after index ischemic event is the period of highest risk of recurrent ischemic events in patients with ICAS (56–59). A consensus conference regarding ICAS held that the risk of recurrent TIA or stroke is highest within 2 weeks after index ischemic event in patients with symptomatic ICAS (60). The WASID (the Warfarin vs. Aspirin for Symptomatic Intracranial Disease) trial showed that patients treated within 2.5 weeks after the first ischemic event had a 1.7-times higher risk of recurrent stroke than those treated later (61). It is seemingly that early endovascular treatment after the index ischemic event is more significant to reduce the risk of recurrent ischemic events in patients with symptomatic ICAS than deferred endovascular treatment. However, angioplasty or stenting itself is a risk factor that may increase the rate of recurrent TIA or stroke due to plaque vulnerability and disorder of blood-brain barrier in initial time period after the index ischemic event. Delaying the procedure for symptomatic patients with recent TIA or stroke may allow stabilization of plaque and cerebrovascular self-regulation, which would offset the adverse aspects of endovascular treatment. The National Institutes of Health Multicenter Wingspan Intracranial Stent Registry Study of 160 symptomatic ICAS patients treated by Gateway balloon and Wingspan stent system demonstrated that stent placement performed 10 days after a qualifying ischemic event was associated with a lower rate of 30 days stroke and/or death compared with stent placement performed within 10 days of the event (8 vs. 17%, *P* = 0.082) (46). Several Asian intracranial stenting trials excluding patients of acute stroke within 3 weeks concluded a low risk of recurrent stoke or death in patients treated with angioplasty or/and stenting (e.g., Miao 4.3%; Gao 2%) (12, 13). Giving in positive data from Asian trials and our anecdotal experience, a time interval of 3 weeks from the qualifying event to endovascular treatment may be a proper cutoff that has a greater benefit for symptomatic ICAS patients, certainly, which requires further studies.

### Type of angioplasty and stenting

**Figure 4:**
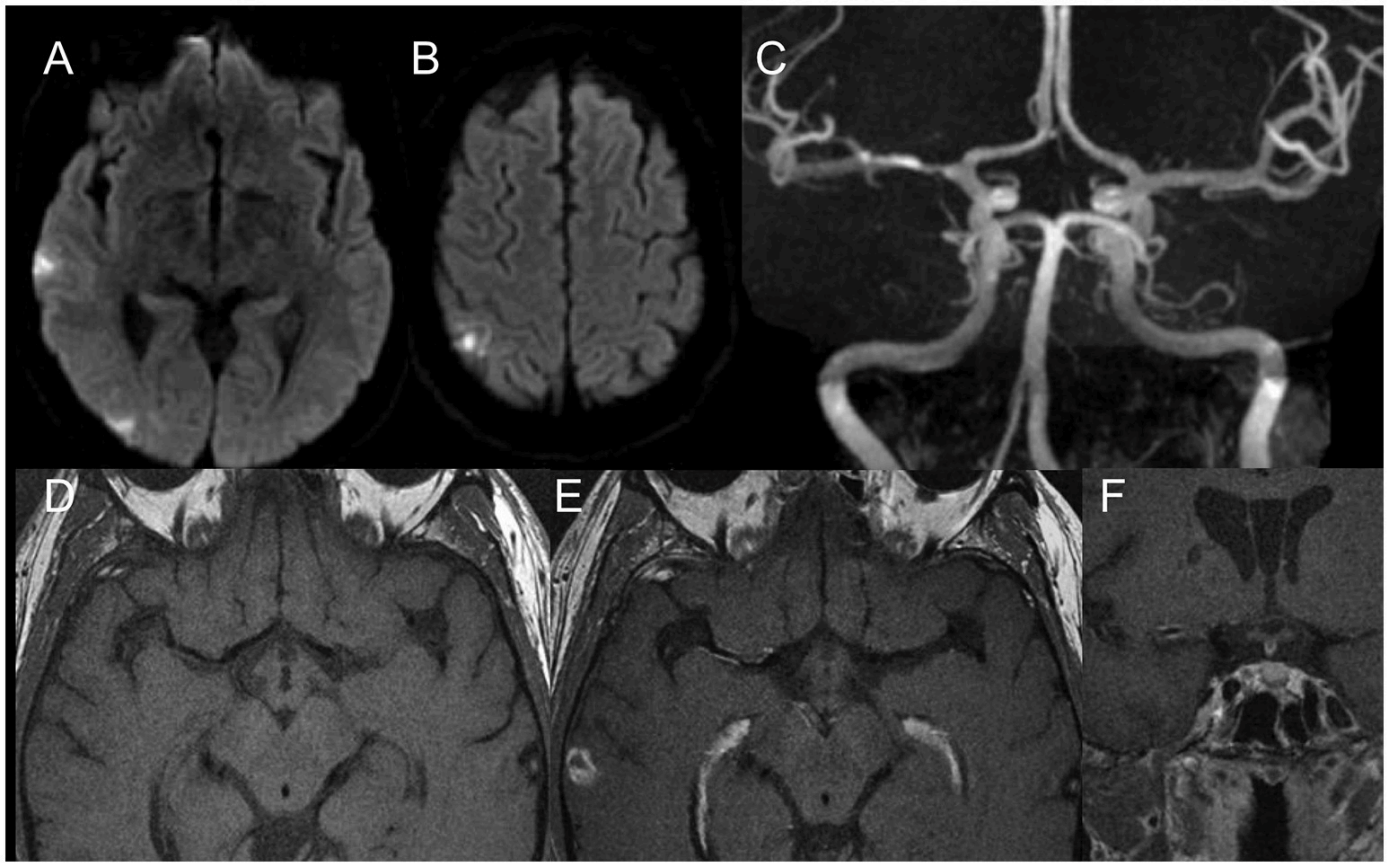
Diffusion weighted scans **(A,B)** demonstrate multiple distal (embolic) foci of ischemia in the right MCA territory. MR Angiography **(C)** shows a moderate degree MCA stenosis in the proximal M1. High resolution vessel wall imaging in axial cuts before **(D)** and after **(E)** contrast enhancement as well as coronal T1 weighted vessel wall imaging sequences after contrast enhancement **(F)** demonstrate a hot plaque with dense eccentric enhancement. Given the embolic nature, the “hot plaque” characteristics and the relatively low degree of stenosis in a patient who was not on optimal therapy, it was decided to not perform an endovascular therapy.

Endovascular technologies are constantly evolving with the development of new technologies of stent deployment and delivery. Next generation stents are likely to be more flexible, easier to deliver, and capable of preventing long-term restenosis. However, the contemporary design of endovascular treatment for ICAS, such as balloon angioplasty alone, self-expandable stents, and balloon-mounted stents, has inherent advantages as well as disadvantages. Based on the characteristics of the plaque, procedural arterial access, length of lesions, and diameter of culprit arteries, different types of endovascular treatment may be chosen individually to obtain the best possible clinical outcomes. According to the Mori classification, three different types of stenoses can be subclassified: Mori A, a short and concentric lesion with a short length (< 5 mm); Mori B, a tubular or extreme eccentric lesion with intermediate length (between 5 and 10 mm); Mori C, a diffuse lesion with a long length (>10 mm) (62). These authors argues, that balloon-mounted stents are suitable for patients with smooth arterial access and Mori A lesions, midbasilar artery, and distal M1 segment lesions; self-expanding stents may be suitable for patients with tortuous arterial access and Mori B or C lesions; and balloon angioplasty alone is suitable for patients with tortuous arterial access with Mori A lesions and a small target-vessel diameter of < 2.5 mm (11). In addition, characteristics of plaque can now be identified using high-resolution vessel wall magnetic resonance imaging (HR-MRI) for detailed visualization of the vessel wall before endovascular treatment (see Figure 4 for an example) (63–65). For example, ulcerous plaques, fibrous cap ruptured plaques, or plaques adjacent to perforator-rich vessel segments can be identified by HR-MRI to help clinicians make better clinical decisions and risk assessments regarding ICAS treatment (66).

### Learning curve of intracranial angioplasty or stenting

Because endovascular treatment requires operators to undergo a learning process, perioperative complications may be reduced by improving operator experience (67). A prospective study of 95 consecutive patients at a single center, splitting data into quarters for learning curve analysis, demonstrated that procedural problems, technical failures, and guidewire- or angioplasty-related hemorrhage were almost the same in the first three quarters but significantly declined in the fourth quarter, indicating a learning curve and a trend of technical maturation in the fourth quarter (47). As there is a learning curve to achieve technical maturity, operators are required to learn from their own mistakes in previous practice and meanwhile to absorb experience from other operators and the literature (41). In addition, alternative training techniques, such as simulation models and virtual reality training, have become valid approaches for training interventionalists (68). Using these techniques, the operator's experience for endovascular treatment could be enhanced in order to maximally guarantee the safety of patients and the efficacy of the endovascular therapy.

As literatures reported, the measures to assess experience of operator for endovascular treatment include: (a) individual accumulative cases of intracranial angioplasty or/and stenting for ICAS in total; (b) individual mean cases of intracranial angioplasty or/and stenting for ICAS per year; (c) the morbidity or mortality rates of angioplasty or/and stenting for ICAS submitted by individual; and d) the center volume of angioplasty or/and stenting cases for ICAS per year (48, 69, 70). Unfortunately, there is a lack of consensus to evaluate technical maturity for operator due to the diversity of interventional discipline and medical condition around the world. We suggest a combination of four measures mentioned above be used to assess the technical maturity for endovascular treatment. For instance, a pilot study of China Angioplasty and Stenting for Symptomatic Intracranial Severe Stenosis (CASSISS) trial was performed to test the credentialing of the operators and participating centers from three aspects of stenting experience, perioperative complications, and the volume of stenting cases. The study demonstrated an excellent result of endovascular treatment for ICAS that only two ischemic strokes within 30 days (13, 70).

## Conclusions

Endovascular treatments, such as balloon angioplasty alone, balloon-mounted stents, and self-expandable stents, may be of benefit for carefully selected ICAS patients. Two prospective, multicenter, RCT are presently underway to re-evaluate the benefits of endovascular treatments in carefully selected patients (CASSISS trial, and the Wingspan Stent System Post-Market Surveillance Study (WEAVE) trial) (70, 71). These trials' strict selection criteria, identification of stroke mechanisms of intracranial atherosclerosis, as well as use of experienced neurointerventionists in high-volume centers are what makes them of interest for the re-evaluation of invasive ICAS treatment.

## Author contributions

LJ and PG provided ideas of the review. JL conducted the review and drafted the initial manuscript. TK and TW critically reviewed and revised the review. All the authors reviewed and approved final version of the manuscript.

### Conflict of interest statement

The authors declare that the research was conducted in the absence of any commercial or financial relationships that could be construed as a potential conflict of interest.
